# An exploration of applicant perceptions of asynchronous video MMIs in medical selection

**DOI:** 10.15694/mep.2018.0000285.1

**Published:** 2018-12-14

**Authors:** Lara Zibarras, Fiona Patterson, Jessica Holmes, Charlotte Flaxman, Angela Kubacki

**Affiliations:** 1 City; 2 Work Psychology Group; 3 St George's

**Keywords:** Selection, Applicant fairness perceptions, Asynchronous video interviewing, MMI

## Abstract

This article was migrated. The article was marked as recommended.

Over the last two decades, technological advancements internationally have meant that the Internet has become an important medium for recruitment and selection. Consequently, there is an increased need for research that examines the effectiveness of newer technology-mediated selection methods. This exploratory research study qualitatively explored applicant perceptions of fairness of asynchronous video interviews used in medical selection. Ten undergraduate medical students participated in a pilot asynchronous multiple-mini interview and were invited to share their experiences and perceptions in a follow-up interview. The data was transcribed verbatim and analysed using template analysis, with Gilliland’s (1993) organisational justice theory guiding the original template. Many of the original themes from Gilliland’s model were uncovered during analysis. Additionally, some significant themes were identified that did not form part of the original template and were therefore added to the final coding template - these were specifically relating to technology, including acceptability in a medical context; technical issues and adverse impact. Overall, results suggested that participants perceived asynchronous video interviews to be a fair method of selection. However, participants thought asynchronous interviews should only be used as part of an extensive selection process and furthermore, should not replace face-to-face interviews. Findings are discussed in line with existing research of fairness perceptions and justice theory in selection ( Gilliland, 1993) and implications for research and practice are presented.

## Introduction

Over the last two decades the Internet has emerged as an important medium for recruitment and selection, from pre-selection, online applications, web- or mobile-based psychometric tests, through to interactive work sample tasks ( Toldi, 2011; Konradt, Warszta and Ellwart, 2013; Gelinas *et al.*, 2017). However, there is still relatively little in the academic literature examining applicant perceptions of technology or web-based selection in healthcare or otherwise, despite its proliferation in practice.

Recent technological advancements have revolutionised the interviewing process so that face-to face interaction is viewed by some as no longer necessary ( Levashina *et al.*, 2014). Telephone interviews are commonly-used across organisations ( Straus, Miles and Levesque, 2001), and videoconferencing tools, such as FaceTime and Skype, are becoming popular and efficient alternatives to face-to-face interviews ( Daram, Wu and Tang, 2014). Also known as *synchronous video interviews,* they are cost effective, and may be preferable to telephone interviews because the interviewer and applicant can see, as well as, hear each other ( Langer, König and Krause, 2017).

More recently, *asynchronous video interviews* (AVI) have been developed so that the interaction between the applicant and the interviewer does not have to occur at the same time ( Levashina *et al.*, 2014). Asynchronous video interviews enable applicants to record their answers to predetermined questions at their leisure ( Brenner, Ortner and Fay, 2016) and these responses can later be reviewed by an assessor ( Torres and Gregory, 2018).

Asynchronous video interviews have several benefits for the applicant and the organisation alike: first, they do not require the presence of organisational members during the interview, so may be more time and cost effective than face-to-face interviews ( Mejia and Torres, 2018). Second, since there is no need to schedule interviews, applicants can complete the interview at a convenient time and place, and the interviewer can review the responses in the same way ( Langer *et al.*, 2017). Third, responses are recorded, so they can be interpreted by multiple assessors which may reduce interviewer bias and improve reliability ( Langer *et al*., 2017). Finally, there is no opportunity for interviewers to digress from the questions, which may improve the validity of asynchronous video interviews ( Chapman and Zweig, 2005).

### The use of Multiple Mini Interviews in Medical Selection

One popular way of assessing non-academic attributes within medicine is through a Multiple Mini Interview (MMI) ( Patterson *et al.*, 2016). The MMI is split into several different “stations”, which may take the form of a question, a simulation, or a presentation (varying in duration, number of assessors, and methods of rating; Eva *et al.*, 2009). The stations may be virtual, i.e. completed online, or they may be physically represented by a number of different tables in an interview room. Since an MMI gives the opportunity to assess an individual in different scenarios, they are likely to be a stronger predictor of future job performance than traditional panel interviews ( Eva *et al.*, 2009). Research shows that MMIs (in video format) can save 84% in recruitment costs for graduate medical and dental programmes and are likely to be as reliable and valid as in-person MMIs since no significant differences were found in applicant scores between both interviews ( Tiller *et al.*, 2013).

Understanding the effectiveness of selection methods is important within medicine ( Patterson *et al.*, 2012) since it is a high profile process subject to scrutiny from a range of key stakeholders. There is an ethical need to ensure the most appropriate students are selected (from the point of view of the public); and applicants have likely invested years of time, money and effort to pursue their future career and thus have a lot at stake with the outcome of a selection method ( Patterson *et al.*, 2012). Therefore, perceptions of fairness are critical in medical selection.

### Fairness perceptions

Fair selection methods can be seen from two perspectives. First, selection methods must be valid and reliable to meet equal opportunities legislation requirements. Second, the applicants’ perspective is important ( Patterson and Zibarras, 2011; Patterson *et al.*, 2012). If applicants do not perceive selection methods to be fair there can be negative implications for the organisation ( Patterson *et al.*, 2011). Negative reactions may result in the loss of good applicants from the selection process ( Kelly *et al.*, 2014; McLarty and Whitman, 2016); or costly legal challenges ( Schmitt and Chan, 1999), and can have a knock-on effect of negative publicity if applicants who perceive the process as unfair actively dissuade other applicants, reducing the total number or quality of the applicant pool ( Anderson, 2011; McCarthy, Hrabluik, & Jelley, 2009; McLarty & Whitman, 2016; Patterson et al., 2011).

Within the wider selection literature, the dominant model for research on applicant reactions is presented by Gilliland ( 1993, 1995) who proposes organisational justice theory ( Greenberg, 1987) as a framework to consider applicant reactions (see Table 1). Variability has been found in the perceived fairness of different selection methods where several studies have compared cross-national fairness perceptions for 10 common selection methods (interviews, CVs, work samples, biodata, ability tests, references, personality questionnaires, honesty tests, personal contacts and graphology; e.g. Steiner and Gilliland, 1996; Moscoso and Salgado, 2004; Anderson and Witvliet, 2008; Hoang *et al.*, 2011). Findings indicate a relatively stable pattern of results where interviews have been consistently rated most favourably.

**Table 1.** Procedural justice rules underlying perceptions of selection processes ( Gilliland, 1995)

Rule	Description
Job relatedness	The extent to which selection methods appear to measure content that is relevant to the job role or appears to be valid
Opportunity to perform	The extent to which applicants can demonstrate their knowledge, skills and abilities
Reconsideration opportunity	Applicants having the opportunity to review test results or challenge scores, or to be able to re-test
Administration consistency	The degree to which selection processes are consistent or standardised across people and over time
Feedback	The provision of feedback that is timely and informative regarding test results and selection decision
Selection information	Information on, and justification for, the use of selection methods and decisions made
Honesty	The extent to which communication with applicants is candid
Interpersonal effectiveness	The extent to which applicants are treated with respect during the selection process
Two-way communication	The opportunity for applicants to ask questions during interpersonal interaction throughout the selection process
Propriety of questions	The degree to which the questions asked of applicants during the selection process are appropriate

However, there is a dearth of research focused on newer technology-mediated selection methods, so little is known about their perceived fairness ( Langer, *et al.*, 2017). Although a recent meta-analysis (of the few studies that exist) suggested that technology-mediated interviews are perceived as less fair than face-to-face interviews ( Blacksmith, Willford and Behrend, 2016) mainly due to the restricted ability to use impression management techniques (gestures and nodding), to influence positive interview performance ( Toldi, 2011). However, asynchronous video interviews were not included in this analysis, which further highlights the need for research.

When considering AVIs, there may be some issues that would result in more negative reactions. For example, applicants may worry about how technical glitches could impact their performance in an AVI ( Brenner, *et al.*, 2016). Additionally, the lack of presence of an interviewer ( Langer *et al*., 2017); the ‘impersonal feel’, and no opportunity to ask for feedback ( Guchait *et al.*, 2014) have all been cited as possible reasons for negative reactions. These characteristics of AVIs suggest that some of the “fairness perception” rules in Gilliland’s (1993) model may not be fulfilled (i.e. little opportunity for two-way communication; less opportunity to perform, etc).

### The Present Study

To the best of the authors’ knowledge, there is currently no research that explores applicant perceptions of AVIs within medical selection. As there is relatively little known about this selection method, a qualitative study is likely to reveal novel insights regarding how fairness is defined within medical selection, and specifically in relation to AVIs. Therefore, we posed the following exploratory research question: *To what extent do medical students perceive asynchronous video interviews to be a fair selection method?*

## Methods

### Participants

The sample comprised 10 undergraduate medical students (three second year students, one third year, and six fourth years) from St George’s, University of London. There were 8 female and 2 male participants, aged between 20 and 28.

### Data collection: semi-structured interviews

A semi-structured interview process was used to allow a deep exploration of the topic, with further probing if necessary ( Willig, 2013). The questions were designed to be open-ended and to allow for as much free-flowing conversation as possible, whilst ensuring minimal input from the interviewer ( Smith and Osborn, 2015). An example question (with probing questions) was:

• *How fair did you find the asynchronous interview process that you undertook?*
  • *What aspects made it fair?*
  • *Are there any aspects that could be changed to make it fairer?*

To gain the most accurate overview of the applicant experiences, the interviews were arranged within 48 hours of completing the asynchronous interview. Data collection interviews took place over the phone, to allow flexibility around the participants’ schedules, during August 2018. To ensure consistency, the same interviewer conducted all interviews, ranging from 23 and 34 minutes.

### Procedure

All participants completed an MMI using an asynchronous video-interviewing platform. The participants were within their first to fourth year at medical school and therefore whilst they were not explicitly completing the interview as part of a selection process, they were told to consider it as a selection process for a medical training post. The MMI was desgined using best practice methodology (comprised six stations that had been designed to assess some of the key non-academic competencies of a doctor e.g. empathy and communication; Patterson et al., 2013). Participants were sent an email to invite them to complete the AVI over any 30-minute period during one week from invitation, using a mobile phone or a computer. Within the email invitation there were instructions for how to complete the AVI, including a link to access it, how long they would have to complete it and that they would be contacted afterwards for a follow-up interview with one of the research team to discuss their perceptions of the AVI process. Participants were also given the opportunity to answer a practice question before they began.

Sixteen medical students responded to the invitation to be interviewed about their experiences of the MMI, however only 10 participants completed the asynchronous MMI within the designated time frame.

Ethics approval for this study was granted by City University of London’s psychology research ethics committee. Participants were sent some background information about the study’s aims and were required to sign and return a consent form in order to take part. Participants took part voluntarily, could withdraw at any point, and gave their consent to interviews being audio recorded. They were assured that their responses/identity would remain anonymous, and interview recordings would remain confidential. Interviews were transcribed verbatim and all names were removed to ensure confidentiality of the participants.

### Data Analysis Procedure

Analysis of the transcripts followed the template approach described by Crabtree and Miller (1999). This involves constructing a coding template with codes representing themes identified in the data through meticulous reading of the text. Codes are organized hierarchically so that the highest level codes represent broad themes in the data, with lower levels indexing more narrowly focused themes. It is normal in template analysis to define *a priori* a number of themes that reflect areas identified as particularly salient to the aims of the research project ( Willig, 2013).

In this study the initial template was based on Gilliland’s (1993) procedural justice rules and the initial template is presented in Table 2. For the analysis, all data was read through and where segments corresponded to *a priori* themes, they were coded as such. Otherwise, new themes were defined to include the relevant material and organised into an amended template. The amended template was then applied to the whole data set, and constantly modified after consideration of each transcript. The final version is presented in Table 3. The template serves as the basis for the interpretation of the data set.

**Table 2.** Original template used for the coding of the interview

1.	Formal characteristics of the process	1.1 Job-relatedness 1.2 Opportunity to perform 1.3 Reconsideration opportunity 1.4 Administration consistency
2.	Explanation/information offered to applicant	2.1 Feedback on performance 2.2 Selection process information 2.3 Honesty in treatment
3.	Interpersonal treatment	3.1 Recruiter effectiveness 3.2 Two-way communication 3.3 Propriety of questions

**Procedural justice rules**

## Results/Analysis

The interviews produced rich, complex and often lengthy accounts of participants’ experiences. Many of the original themes from Gilliland’s model were uncovered during analysis. Additionally, some significant themes were identified during coding that did not form part of the original template and were therefore added to the final coding template (see Table 3). Here we briefly outline the final coding template with illustrative quotes.

**Table 3.** Final Coding Template

1.	Formal characteristics of the process	1.1 Job relatedness 1.2 Opportunity to perform 1.2.1 Variety of Skills 1.2.2 Opportunity to Cheat 1.3 Reconsideration opportunity 1.4 Consistency of administration
2.	Explanation/information offered to applicant	2.1 Feedback on performance 2.2 Selection process information 2.3 Honesty in treatment
3.	Interpersonal treatment	3.1 Recruiter effectiveness 3.1.1 Multiple assessors 3.2 Two-way communication 3.3 Propriety of questions
4.	Technology	4.1 Acceptability in medical context 4.2 Convenience 4.3 Technical issues 4.3.1 Adverse impact

### Formal Characteristics of the process

#### Job Relatedness

Job-relatedness was a major determinant of participants’ fairness perceptions, as was assessing both academic *and* non-academic qualities in order to generate a job-related overview of a person.

“A huge part of medicine is about social skills and your communication skills.. human interaction and your ability to interact with patients.. so I think if you’re not assessing these, an interview is not representative of medicine” (P4)

#### Opportunity to perform

This related to the adequate opportunity to demonstrate knowledge, skills, and abilities relevant to the job.

“..to ensure that everyone has a fair chance to shine” (P8)

During the AVI, several participants felt that they would not have the opportunity to fully express themselves, or use body language to enhance their performance:

“Whereas in a normal interview.. you’d be able to get like all of your body language and your hand movements across” (P4)

Conversely, some participants indicated that because the interviewer is not present, it would make the process more objective:

“You know you won’t be judged by.. interviewers, seeing the way you’re acting beforehand or you know you’re standing nervously outside each station and they might judge you based on that rather than your answers” (P10)

##### Variety of skills

Within opportunity to perform, applicants felt that a fair selection method would assess a wide range of qualities so that a three dimensional overview of the applicant’s skills is gathered.

“The whole dimensions of what a person is doing, their mannerisms and patience and everything” (P9)

##### Opportunity to cheat

On the other hand, there were concerns that the preparation time given at the start of each question may present an opportunity for cheating, and enable some participants to gain an unfair advantage.

“Is there room to get.. help from someone? In that minute could I have a discussion very quickly with someone about the points I needed to say because that one minute is not live” (P8)

#### Consistency of administration

Participants agreed that the AVI was a fair selection method because all applicants undergo the same process, and it is consistent and objective for everyone. For example, all applicants are asked the same questions and then assessed in the same way:

“Interview all applicants equally, like on an equal basis, with all factors being the same except.. the quality of their answers that they give” (P9)

“The fact everyone’s given the same time slot, the same layout” (P7)

Participants also identified that the technology meant that everyone had the same experience because there was no opportunity to digress from standardised questions.

“You can’t go off in tangents in conversation it’s just the questions you’re being asked” (P3)

### Explanation

#### Feedback

One element that participants felt was not fair about the AVI process, was that because the process was automated, you do not get immediate feedback:

“..always like to get feedback.. from the interview or how it went .. because obviously with these kind of things.. compared to live interviews where you talk to a person, and they say thank you this will happen, or this will happen.. you’ll get feedback on your interview or something within the next few days” (P1)

#### Selection information

A further issue that participants noted, was the lack of information about the selection method and process:

“..if you had given me a bit more information about what you were going to ask me I would be better prepared” (P2)

“..you’d want to know what happens to the interview after this.. who’s going to be viewing it, how many people are going to view it, what time scale is it going to be viewed in, etc, might be quite useful information to have” (P3)

### Interpersonal Treatment

#### Recruiter Effectiveness

Participants mentioned that the process felt fairer because applicants were able to gain a balanced view of the selection process, compared to when participants might encounter an ‘ineffective’ recruiter:

“Where the interviewers are not passionate about the role themselves.. if they’re just there because it was their turn on the rota to interview some medical students then they’re not going to be engaging” (P3)

##### Multiple assessors

Participants indicated that the more assessors involved in the rating of the interviews, the fairer the interview would be.

“The more people that you get it means that that subjectivity is reduced a little bit and at least you get the opinions of a few people.. I assume these are respected people who know what they’re talking about” (P5)

#### Two-way communication

The majority of participants disliked the lack of an interviewer’s presence and found it unfair in some instances.

“When you have a real life interview it’s nice to interact with the interviewer..whereas with this..nobody was listening to you actively.. I’ve never done anything like that before and it is hard to just talk when nobody is listening to you” (P10)

“if I had an interviewer, a real person asking me questions.. then it would be easier for me to give feedback” (P1)

#### Acceptability of the Use of Technology in a Medical Context

Participants discussed how using AVIs was acceptable in the medical context. The majority of participants agreed that advances in technology meant that AVIs are likely to become more commonplace.

“I think this is the future.. we will see a lot more of it if some people agree to it or not”. (P10)

In addition, participants agreed that AVIs have a place within a fair selection process in medicine. However, importantly, participants also felt that they should not be used as a replacement for face-to-face interviews, and never the sole determinant of a job offer.

“It’s definitely a good part, to do as part of the actual process, but I’m not sure how confident I’d be if it was just the whole thing relied on the asynchronous interview” (P1)

#### Convenience

Participants felt that the technology enabled a fairer process because applicants could complete the interview at a time and place convenient for them.

“You can just do it wherever you want as long as it’s a quiet place and somewhere that’s convenient for you” (P10)

Convenience also seemed to “soften the blow” if the applicant is unsuccessful:

“The candidates might be travelling across the country to just interview and go back and..if their application is unsuccessful then they’ve kind of wasted their time” (P10)

#### Technical issues

Concerns were raised surrounding the use of technology, where several participants said they would worry whether technical issues may hinder their performance.

“You have the worry that a technical problem might happen on the day and then that will change your interview” (P9)

##### Adverse impact

Some participants also remarked that applicants might be disadvantaged if they are less used to this type of technology:

“Someone who’s not very technically au fait or slightly older candidates or something like that, they might struggle with, if they’ve got an old laptop or something” (P5)

## Discussion

This exploratory study examines applicant fairness perceptions of asynchronous video interviews in medical selection. Using template analysis, findings suggested that on the whole, applicants perceive AVIs to be fair. In line with existing research ( Hausknecht, Day and Thomas, 2004), participants emphasised this selection method would clearly assess the skills that are relevant to the role of a doctor.

Interestingly, many of Gilliland’s justice rules were discussed by applicants, so in terms of examining the validity and importance of Gilliland’s rules, this study found that many were relevant, but some were less salient than others. In particular, there were three elements of justice that were not discussed at all. These were: *reconsideration opportunity, honesty* and *propriety of questions.*

Nevertheless, many of the justice rules were salient, along with other important categories. The importance of selecting applicants for medicine based on non-academic as well as academic skills is widely recognised within research ( Patterson, Cleland and Cousans, 2017). Participants also felt that fair selection methods would examine a variety of non-academic and academic skills, and that competent clinicians require a number of different competencies beyond academic attainment.

Although participants felt that AVIs would give them adequate opportunity to perform, there was a concern that the preparation time at the start of each question may present an opportunity for cheating. This issue has been explored in e-learning contexts where asynchronous video examinations are widely used to evaluate students’ performance ( Datsenka, Stankov and Kurbel, 2012). In this context, there are practical steps that educators have taken to minimise this risk, for example, asking applicants to video a 360 panoramic view of the room before the examination starts to ensure no-one else is in the room. This could be implemented in selection contexts too.

One element of AVIs that all participants considered fair and discussed at length, was their consistency of administration. AVIs are designed in such a way to ensure that the process (e.g. length of interview and questions asked) is the same for everyone ( Toldi, 2011). There was also an expectation that fairness was improved because multiple assessors could later assess the interviews leading to a fairer and potentially less biased evaluation of the applicant ( Langer *et al*., 2017).

The technology element of AVIs appeared to be a “double edged sword” when applicants discussed perceptions of fairness. On the one hand, participants discussed the AVI’s practical convenience which also meant that they might feel less negatively about a rejection if they have not spent time or money on travelling to an interview. Findings support previous research ( Langer *et al*., 2017) suggesting that applicants would feel more relaxed completing an interview at a time and place of their choice, leading to enhanced performance. Indeed, test-taking anxiety is known to negatively impact performance in selection more generally ( Mccarthy *et al.*, 2013), so steps taken to reduce anxiety are positive.

By contrast, the fear of technical glitches and/or losing parts of the interview may serve to increase anxiety, which supports findings by Brenner *et al* (2016). Participants also identified concerns for some applicants who may be less confident in using technological software, such as a webcam or microphone, and therefore may be disadvantaged. This corroborates research suggesting that a *digital divide* ( Roth *et al.*, 2016) may exist in terms of access to computers and the Internet, where older applicants for example may have less ability and/or capability to access these.

Other issues such as the lack of a ‘personal touch’ were felt to reduce perceptions of fairness. Again, supporting previous research suggesting that technology would prevent applicants from using impression management ( Guchait *et al.*, 2014; Langer, *et al.*, 2017) and the lack of opportunity to build rapport and create a relationship with recruiters (Sears *et al.*, 2013).

Despite this, findings suggested that overall participants were positive about the use of AVIs in a medical education context, and felt that they were fair, so long as they did not fully replace a face-to-face opportunity for interaction. These findings support existing research that suggests that although technology is becoming more commonly used in selection, face to face interaction is still preferred over technology mediated interviews ( Blacksmith *et al*., 2016).

### Theoretical and practical implications

There are several noteworthy implications of this research. Theoretically, study findings highlight the extent to which Gilliland’s (1993) model is idiosyncratic, where perceptions of rule violation and fairness vary across individuals. What is considered to be a rule violation by one individual can be positively viewed by another. It is therefore important to evaluate new selection methods from the applicants’ perspective and examine the extent to which Gilliland’s theory is applicable in this context. Elements are still very relevant today, but may need to be updated in light of such technological advances. This exploratory study is a first step towards understanding applicant perceptions of asynchronous video interviews in a medical education context, and further research could explore this model in different healthcare contexts.

The results of this study provide a number of practical implications for the use of AVIs in medicine. The research highlighted many ways in which AVIs perceived as fair, however some important challenges were raised that would need to be addressed to ensure positive fairness perceptions in any applied context. The first implication relates to the design of the AVIs. It is important that AVIs are job-related ( Zibarras and Patterson, 2015) and measure important competencies relevant to a competent doctor or healthcare professional ( Patterson, *et al.*, 2017).

Second, the information provided to applicants is critical to ensure that they understand why an AVI has been chosen, justification of its use, details of the competencies measured and to highlight its relevance to the role ( Mccarthy *et al.*, 2013). It could also include a list of the advantages for its use so that applicants can understand the potential benefits for them such as time and cost efficiency and reduced bias due to the standardisation of the process.

Third, organisations must take action to mitigate any potential technical issues associated with the use of their chosen video interviewing software ( Datsenka, *et al.*, 2012). For example, organisations must define the conditions for re-taking the interview in cases of technical failure; there must be clarity around what happens should there be connectivity issues, faulty webcam, microphone (and so on).

Fourth, confidentiality issues should be explained, since transmission of personal data may raise concerns around privacy or confidentiality ( Konradt, Warszta and Ellwart, 2013). Finally, since web-based selection requires that applicants use their own equipment, with potential differences in speed of access to the Internet, screen size, and ease of use (Kondradt *et al*., 2013), then usability testing is critical on all platforms and mediums (PC versus mobile) to ensure accessibility for all applicants.

### Limitations and directions for future research

There are some limitations to this study that should be noted. The sample size was small using a single-site medical student population. Therefore, it is important to exercise caution when generalising the findings to other healthcare settings ( Brenner et al, 2016). For example, it could be the case that the challenges associated with the use of AVIs may be less salient outside of this medical context. As competition for places into medical school and postgraduate training is very high, the pressure during selection is heightened for applicants ( Irish *et al.*, 2011). Therefore, it is likely that negative factors, such as opportunities to cheat, may be far more salient than in other settings. It may be that in contexts where selection is lower stakes ( Patterson *et al.*, 2012), AVIs could be a suitable replacement for a face-to-face interviews.

## Conclusion

In conclusion, this study is one of the first to investigate applicant fairness perceptions of AVIs in medicine. Results showed how applicants may perceive AVIs to be a fair method of selection in medicine. Several challenges associated with asynchronous interviews were identified and suggestions were made in order to mitigate their occurrence. In order to ensure positive fairness perceptions, it is important to note how asynchronous interviews should be used in medicine. Participants agreed that asynchronous interviews should only be used as a selection method as part of an extensive selection process within medicine. The findings also provide a support for Gilliland’s (1993) procedural justice rules and show how they can apply to technology-mediated selection methods used today. To conclude, this research study provides useful insights into applicant perceptions of fairness selection methods in medicine, whilst also contributing valuable knowledge to the understanding of a currently under researched selection method.

## Take Home Messages

• Recent technological advancements have revolutionised interviewing, so that face to face interaction is no longer necessary. Asynchronous video interviewing (AVI) means that the interaction between applicant and interviewer does not occur at the same time.
• Multiple Mini Interviews can be carried out in asynchronous video format, yet little research has been conducted to examine applicant fairness perceptions of these.
• Overall, applicants perceived the MMI in asynchronous video format to be fair, particularly around their consistency of administration and job relatedness. There was also an expectation that web-based technologies would increasingly be used in healthcare selection contexts.
• To ensure fairness perceptions, organisations must provide information to candidates about why AVIs are chosen and must take steps to mitigate any potential technical issues that may arise.

## Notes On Contributors

**Lara Zibarras PhD AFBPsS** is Senior Lecturer in Organizational Psychology at City, University of London. As a Chartered Occupational Psychologist and Associate Fellow of the British Psychological Society, Lara has conducted extensive selection assessment research in high-stakes settings, focusing on the applicants’ perspective, diversity and use of innovative assessment methods. She has published widely in academic journals and consulted for public and private sector organisations in the areas of selection, training, development and psychometric assessment. ORCID: https://orcid.org/0000-0002-9522-1679

**Fiona Patterson PhD, CPsychol, MRCGP (Hon)** is a leading expert in the field of selection and assessment in organisations. She is the founding Director for Work Psychology Group, providing advice to public and private sector organisations internationally. She is a Visiting Professor to City, University of London, and the University of Nottingham. Fiona publishes regularly in the highest-ranking journals in medical education and psychology. She co-Chairs INReSH (the international network for researchers in selection into healthcare) and is lead editor for a recently published a book, Selection and recruitment in healthcare; Research, theory and practice (2018). She is currently the lead editor for the MedEd Publish special issue on selection and recruitment in medical education. ORCID https://orcid.org/0000-0002-1031-130X

**Jessica Holmes BSc MSc** has recently completed her MSc in Organisational Psychology at City University. This paper was based on a study she conducted as part of her dissertation research during an internship at Work Psychology Group. She has conducted research in selection, assessment and human behaviour change. She is currently working as a client success manager for Capp, specialising in delivering strengths-based selection assessment and development solutions.

**Charlotte Flaxman BSc MSc** is a Senior Consultant at Work Psychology Group with expertise in selection, assessment and development for high stakes professions. She has designed, implemented and evaluated a range of research led high stakes assessment and development solutions for a wide variety of roles, providing demonstrable benefits in terms of both effectiveness and efficiency for organisations. In particular, Charlotte has significant experience and expertise in the implementation and evaluation of selection tools within medicine, dentistry and pharmacy.

**Angela Kubacki MSc FHEA AFBPS** is a Senior Lecturer in Clinical Communication at St George’s, University of London and is also Associate Dean for Admissions. As a Chartered Health Psychologist and Associate Fellow of the British Psychological Society, she has been involved in clinical education for nearly 20 years and has been working with medical schools admissions teams to develop fair and transparent interview processes. She presented her work on the development of video-based situational judgement analysis stations to assess for professionalism and honesty in MMIs at AMEE 2016 in Barcelona. She is a member of the UKCAT Board of Directors and the Medical Schools Council Selection Alliance and is co-chair of the MSCSA MMI subgroup.

## Declarations

Fiona Patterson is a guest Theme Editor for the theme of Selection and Recruitment in Medical Education in AMEE MedEdPublish.

## References

[ref1] Anderson N. R. (2011) Perceived job discrimination (PJD): Toward a model of applicant propensity to case initiation in selection. International Journal of Selection and Assessment. 19(3), pp.229–244. 10.1111/j.1468-2389.2011.00551.x

[ref2] Anderson N. R. and Witvliet C. (2008) Fairness reactions to personnel selection methods: An international comparison between the Netherlands, the United States, France, Spain, Portugal, and Singapore. International Journal of Selection and Assessment. 16(1), pp.1–13. 10.1111/j.1468-2389.2008.00404.x

[ref3] Blacksmith N. Willford J. and Behrend T. (2016) Technology in the Employment Interview: A Meta-Analysis and Future Research Agenda. Personnel Assessment and Decisions. 2(1). 10.25035/pad.2016.002

[ref4] Brenner F. S. Ortner T. M. and Fay D. (2016) Asynchronous Video Interviewing as a New Technology in Personnel Selection: The Applicant’s Point of View. Frontiers in Psychology. 7, p.863. 10.3389/fpsyg.2016.00863 27378969 PMC4906051

[ref5] Chapman D. S. and Zweig D. I. (2005) Developing a nomological network for interview structure: Antecedents and consequences of the structured selection interview. Personnel Psychology.pp.673–702. 10.1111/j.1744-6570.2005.00516.x

[ref6] Crabtree B. F. and Miller W. L. (1999) Using codes and code manuals: a template organizing style of interpretation. Doing qualitative research. 2, pp.163–177.

[ref7] Daram S. R. Wu R. and Tang S. (2014) Interview From Anywhere: Feasibility and Utility of Web-Based Videoconference Interviews in the Gastroenterology Fellowship Selection Process. The American Journal of Gastroenterology. 109(2), pp.155–159. 10.1038/ajg.2013.278 24496418

[ref8] Datsenka R. Stankov I. and Kurbel K. (2012) Design and Implementation of Remotely Supervised Video-Based Distance Examinations.in 2012 IEEE 12th International Conference on Advanced Learning Technologies IEEE, pp.208–212. 10.1109/ICALT.2012.94

[ref9] Eva K. W. Reiter H. I. Trinh K. Wasi P. Rosenfeld J. (2009) Predictive validity of the multiple mini-interview for selecting medical trainees. Medical Education. 43(8), pp.767–775. 10.1111/j.1365-2923.2009.03407.x 19659490

[ref10] Gelinas L. Pierce R. Winkler S. Cohen I. G. Lynch H. F. (2017) Using Social Media as a Research Recruitment Tool: Ethical Issues and Recommendations. The American Journal of Bioethics. 17(3), pp.3–14. 10.1080/15265161.2016.1276644 PMC5324729 28207365

[ref11] Gilliland S. W. (1993) The Perceived Fairness of Selection Systems: an Organizational Justice Perspective. The Academy of Management Review. 18(4), pp.694–734. 10.2307/258595

[ref12] Gilliland S. W. (1995) Fairness from the applicant’s perspective: Reactions to employee selection procedures. International Journal of Selection and Assessment. 3(1), pp.11–18. 10.1111/j.1468-2389.1995.tb00002.x

[ref13] Greenberg J. (1987) A taxonomy of organizational justice theories. The Academy of Management Review. 12(1), pp.9–22. 10.2307/257990

[ref14] Guchait P. Ruetzler T. Taylor J. and Toldi N. (2014) Video interviewing: A potential selection tool for hospitality managers - A study to understand applicant perspective. International Journal of Hospitality Management. 36, pp.90–100. 10.1016/j.ijhm.2013.08.004

[ref15] Hausknecht J. Day D. V and Thomas S. C. (2004) Applicant reactions to selection procedures: An updated model and meta-analysis. Personnel Psychology. 57(3), pp.639–683. 10.1111/j.1744-6570.2004.00003.x

[ref16] Hoang T. G. Erdogan B. Truxillo D. M. and Bauer T. N. (2011) Cross-cultural applicant reactions to selection methods: Vietnam and United States. International Journal of. 20(2), pp.209–219.

[ref17] Irish, B., Carr, A., Sowden, D., Douglas, N. and Patterson, F. (2011) Recruitment into specialty training in the UK, BMJ Careers.

[ref18] J. Sears G. Zhang H. H. Wiesner W. D. Hackett R. and Yuan Y. (2013) A comparative assessment of videoconference and face-to-face employment interviews. Management Decision. 51(8), pp.1733–1752. 10.1108/MD-09-2012-0642

[ref19] Kelly M. E. Gallagher N. Dunne F. P. and Murphy A. (2014) Views of doctors of varying disciplines on HPATIreland as a selection tool for medicine. Medical Teacher. 36(9), pp.775–782. 10.3109/0142159X.2014.909012 24804920

[ref20] Konradt U. Warszta T. and Ellwart T. (2013) Fairness Perceptions in Web‐based Selection: Impact on applicants’ pursuit intentions, recommendation intentions, and intentions to reapply. International Journal of Selection and Assessment. 21(2), pp.155–169. 10.1111/ijsa.12026

[ref21] Langer M. König C. J. and Krause K. (2017) Examining digital interviews for personnel selection: Applicant reactions and interviewer ratings. International Journal of Selection and Assessment. 25(4), pp.371–382. 10.1111/ijsa.12191

[ref22] Levashina J. Hartwell C. J. Morgeson F. P. and Campion M. A. (2014) The Structured Employment Interview: Narrative and Quantitative Review of the Research Literature. Personnel Psychology. 67(1), pp.241–293. 10.1111/peps.12052

[ref23] McCarthy J. Hrabluik C. and Jelley B. (2009) Progression Through the Ranks: Assessing employee reactions to high-stakes employment testing. Personnel Psychology. Blackwell Publishing Inc,62(4), pp.793–832. 10.1111/j.1744-6570.2009.01158.x

[ref24] Mccarthy J. M. Iddekinge C. H. Van Lievens F. Kung M.-C. Sinar E. F. (2013) Do Candidate Reactions Relate to Job Performance or Affect Criterion-Related Validity? A Multistudy Investigation of Relations Among Reactions, Selection Test Scores, and Job Performance. Journal of Applied Psychology. 98(5), pp.701–719. 10.1037/a0034089 23937298

[ref25] McLarty B. D. and Whitman D. S. (2016) A Dispositional Approach to Applicant Reactions: Examining Core Self-Evaluations, Behavioral Intentions, and Fairness Perceptions. Journal of Business and Psychology. 31(1), pp.141–153. 10.1007/s10869-015-9405-x

[ref26] Mejia C. and Torres E. N. (2018) Implementation and normalization process of asynchronous video interviewing practices in the hospitality industry. International Journal of Contemporary Hospitality Management. 30(2), pp.685–701. 10.1108/IJCHM-07-2016-0402

[ref27] Moscoso S. and Salgado J. F. (2004) Fairness Reactions to Personnel Selection Techniques in Spain and Portugal. International Journal of Selection and Assessment. University of Santiago de Compostela, Spain; University of Santiago de Compostela, Spain,12(1-2), pp.187–196. 10.1111/j.0965-075X.2004.00273.x

[ref28] Patterson F. Cleland J. and Cousans F. (2017) Selection methods in healthcare professions: where are we now and where next? Advances in Health Sciences Education. 22, pp.229–242. 10.1007/s10459-017-9752-7 28341921

[ref29] Patterson F. Knight A. Dowell J. Nicholson S. Cousans F. (2016) How effective are selection methods in medical eduction and training? Evidence from a systematic review. Medical Education. 50(1), pp.36–60. 10.1111/medu.12817 26695465

[ref30] Patterson F. Lievens F. Kerrin M. Zibarras L. and Carette B. (2012) Designing Selection Systems for Medicine: The importance of balancing predictive and political validity in high-stakes selection contexts. International Journal of Selection and Assessment. 20(4), pp.486–496. 10.1111/ijsa.12011

[ref31] Patterson F. Tavabie A. Denney M. Kerrin M. Ashworth V. (2013) A new competency model for general practice: implications for selection, training, and careers. The British journal of general practice : the journal of the Royal College of General Practitioners. British Journal of General Practice. 63(610), pp.e331–e338. 10.3399/bjgp13X667196 PMC3635579 23643231

[ref32] Patterson F. Zibarras L. Carr V. Irish B. and Gregory S. (2011) Evaluating candidate reactions to selection practices using organisational justice theory. Medical Education. 45(3), pp.289–297. 10.1111/j.1365-2923.2010.03808.x 21299603

[ref33] Patterson F. and Zibarras L. D. (2011) Exploring the construct of perceived job discrimination in selection. International Journal of Selection and Assessment. 19(3), pp.251–257. 10.1111/j.1468-2389.2011.00553.x

[ref34] Roth P. L. Bobko P. Van Iddekinge C. H. and Thatcher J. B. (2016) Social Media in Employee-Selection-Related Decisions: A Research Agenda for Uncharted Territory. Journal of Management. 42(1), pp.269–298. 10.1177/0149206313503018

[ref35] Schmitt N. and Chan D. (1999) The status of research on applicant reactions to selection tests and its implications for managers. International Journal of Management Reviews. Blackwell Publishers Ltd. 1(1), pp.45–62. 10.1177/2049463714541642

[ref36] Smith J. A. and Osborn M. (2015) Interpretative phenomenological analysis as a useful methodology for research on the lived experience of pain. British Journal of Pain. 9(1), pp.41–42. 10.1177/2049463714541642 26516556 PMC4616994

[ref37] Steiner D. D. and Gilliland S. W. (1996) Fairness reactions to personnel selection techniques in France and the United States. Journal of Applied Psychology. [Washington, etc.: American Psychological Association, etc.],81(2), pp.134–141. 10.1037/0021-9010.81.2.134

[ref38] Straus S. G. Miles J. A. and Levesque L. L. (2001) The effects of videoconference, telephone, and face-to-face media on interviewer and applicant judgments in employment interviews. Journal of Management. 27(3), pp.363–381. 10.1177/014920630102700308

[ref39] Tiller D. O’Mara D. Rothnie I. Dunn S. Lee L. (2013) Internet-based multiple mini-interviews for candidate selection for graduate entry programmes. Medical Education. 47(8), pp.801–810. 10.1111/medu.12224 23837426

[ref40] Toldi N. L. (2011) Job applicants favor video interviewing in the candidate‐selection process. Employment Relations Today. Wiley-Blackwell.38(3), pp.19–28. 10.1002/ert.20351

[ref41] Torres E. N. and Gregory A. (2018) Hiring manager’s evaluations of asynchronous video interviews: The role of candidate competencies, aesthetics, and resume placement. International Journal of Hospitality Management. 75, pp.86–93. 10.1016/j.ijhm.2018.03.011

[ref42] Willig C. (2013) Introducing Qualitative Research in Psychology. 3rd edn, Journal of Chemical Information and Modeling. 3rd edn. Open University Press.

[ref43] Zibarras L. D. and Patterson F. (2015) The Role of Job Relatedness and Self-efficacy in Applicant Perceptions of Fairness in a High-stakes Selection Setting. International Journal of Selection and Assessment. 23(4), pp.332–344. 10.1111/ijsa.12118

