# Supplementary material for: An exploration of applicant perceptions of asynchronous video MMIs in medical selection
Source: MedEdPublish (2016). 2018 Dec 14;7:285. [Version 1] doi: 10.15694/mep.2018.0000285.1 (PMC10711982; doi:10.15694/mep.2018.0000285.1)
Supplement: Peer review history

# Reviewer report: Reviewer response for version 1

**DOI:** 10.21956/mep.19731.r29557

**Published:** 2018-12-23

**Authors:** Ken Masters

**Affiliations:** 1 Sultan Qaboos University

This review has been migrated. The reviewer awarded 4 stars out of 5 This is a small but very effective study on the perceptions of students undergoing Asynchronous Video Interviews (AVIs) as part of their selection process. The study is well-structured, and the data are clearly presented. As the authors note, an important limitation is the small sample size, so it is rather a pilot (as they note) more than a full-blown study. Nevertheless, it has useful insights for those involved in student selection, and is a worthy addition to our knowledge of the area.

Reviewer Expertise:

NA

No decision status is available

# Reviewer report: Reviewer response for version 1

**DOI:** 10.21956/mep.19731.r29558

**Published:** 2018-12-16

**Authors:** Ian Wilson

**Affiliations:** 1 International Medical University

This review has been migrated. The reviewer awarded 5 stars out of 5 this is an important and valuable study. the messages arising from the research are important to all involved in medical student selection.The only limitation of the research is that it was conducted on students already accepted. This does not detract from the messages, but does limit the generalisability.An excellent study with important results.

Reviewer Expertise:

NA

No decision status is available
